# Assessment of the Prognostic Value of Monocyte-to-HDL Ratio in ST-Elevation Myocardial Infarction Patients Undergoing Primary Percutaneous Coronary Intervention -

**DOI:** 10.31661/gmj.v12i.3126

**Published:** 2023-12-01

**Authors:** Ahmad Separham, Naser Aslan-abadi, Hamid Sedigh, Reza Javan-ajdadi, Kazem Mehravani

**Affiliations:** ^1^ Cardiovascular Research Center, Faculty of Medicine, Tabriz University of Medical Sciences, Tabriz, Iran

**Keywords:** ST-elevation Myocardial Infarction, Inflammation, Monocyte HDL Ratio, Cardiovascular Events

## Abstract

Background: The purpose of this study was to assess the prognostic value of the monocyte-to-high-density lipoprotein ratio (MHR) as a marker of inflammation in patients diagnosed with ST-segment elevation myocardial infarction (STEMI) who underwent primary percutaneous coronary intervention (PCI).Materials and Methods: This retrospective cross-sectional study was conducted on patients with a diagnosis of STEMI who underwent PCI between March 2021 and March 2022 at Madani Training and Research Hospital in Tabriz, Iran. Data regarding clinical and demographic properties, and laboratory parameters were obtained from medical records. Patients were categorized into two groups according to the median of admission MHR. Results: The study population consisted of 652 patients, 378 males (58%), and 275 females (42%), with a median age of 68 years (interquartile range: 57-77). Results showed that groups with higher MHR (15.59) had higher rates of in-hospital mortality and higher major adverse cardiovascular events (MACEs) in comparison with the group featuring lower MHR (15.59). Receiver operating characteristic (ROC) curves demonstrated that MHR could predict in-hospital mortality with a 75.7% sensitivity and 53.5% specificity, as well as predict MACE with 60.2% sensitivity and 59.7% specificity. Multivariate analyses indicated that MHR is an independent predictor of both in-hospital mortality (OR 1.05, 95% CI 1.02-1.08, P=0.002) and MACE (OR 1.05, 95% CI 1.02-1.08, P0.001). Conclusion: This research indicated that the rise in MHR was independently associated with a higher risk of MACE and in-hospital mortality in STEMI patients undergoing primary PCI.

## Introduction

Coronary artery disease (CAD) is a major health concern leading to over 17.8 million deaths annually [1]. The underlying cause of CAD is atherosclerotic cardiovascular disease, which is associated with acute coronary syndrome (ACS) including ST-segment elevation myocardial infarction (STEMI), non-ST-segment elevation myocardial infarction (NSTEMI), and unstable angina. Atherosclerotic plaque rupture constitutes the major underlying cause of STEMI since its major components are, endothelial cells, leukocytes, and intimal smooth muscle cells [2].

Inflammation and lipid accumulation play a substantial role in the development of atherosclerosis and cardiovascular disease [3]. Chronic inflammation is associated with an increase in inflammatory mediators, cytokines, and adhesion molecules. This inflammatory response is closely related to the formation, progression, and rupture of atherosclerotic plaques [4]. Furthermore, the absorption of low-density lipoprotein (LDL-c) by macrophages in the vessel wall leads to the release of inflammatory cytokines and necrosis of the atherosclerotic plaque [5].

Monocytes play a critical role in the development and progression of atherosclerosis, and their number can be a predictor of heart events [6]. In inflammatory or pro-thrombotic states, monocytes can express tissue factors and become pro-coagulants [7]. Additionally, activated monocytes release circulatory pre-inflammatory cytokines that can damage the elastic layer and cause atherosclerotic plaque rupture [8]. In contrast, high-density lipoprotein (HDL-c) has anti-inflammatory, antioxidant, and anti-thrombotic effects. The low level of HDL-c in patients is associated with an increased hospital mortality risk following myocardial infarction (MI). HDL-c protects endothelial cells from inflammation and oxidative stress by controlling the activation and proliferation of monocytes and preventing their absorption into the artery wall. It has also been shown that HDL-c, as an anti-atherosclerotic agent, can inhibit the expression of tissue factor in monocytes by preventing P38 mitogen-activated protein kinase activation and inhibiting phosphoinositide 3-kinase (PI3K) [9][10]. It is hypothesized that there is a relation between the number of monocytes, the low HDL-c levels, and the progression of atherosclerosis and consequently cardiovascular accidents. Previous studies have found that regression of coronary atherosclerotic plaques after treatment with pravastatin is associated with increases in serum HDL-c and decreases in peripheral blood monocyte counts [11]. This has led to the introduction of the monocyte-to-high-density lipoprotein ratio (MHR) as a novel cardiovascular biomarker that may be more effective than individual measurements of monocyte count and HDL-c levels in predicting both short-term and long-term outcomes of CAD [12][13]. Several studies have shown that MHR during hospitalization in ACS patients is significantly associated with C-reactive protein (CRP) and other inflammatory indices, and patients with higher MHR are at higher risk of in-hospital death, major adverse cardiovascular events (MACEs), and stent thrombosis [7][14][15][16]. Given these findings, this research was designed to assess the correlation between the admission MHR values, in-hospital mortality, and MACE in patients who had experienced STEMI and undergone primary percutaneous coronary intervention (PCI).

## Materials and Methods

This retrospective cross-sectional study analyzed the medical records of patients with a diagnosis of STEMI who underwent primary PCI between March 2021 and March 2022 at Madani Training and Research Hospital, Tabriz, Iran. Patients who presented within 12 hours of the onset of symptoms (typical chest pain lasting for more than 30 minutes) and had ST-segment elevation of 1 mm or greater in at least two consecutive leads were included in the study. Subjects with clinical signs of heart failure (HF), history of MI, active cancer, hematological proliferative disorders, active hepatobiliary diseases, chronic antihyperlipidemic treatment, active infection, chronic inflammatory disease, receiving steroid therapy, autoimmune disease, and those without a recorded measurement of admission laboratory parameters including cholesterol levels before PCI were excluded from this study. In this study, patients were divided into two groups according to the median of their admission MHR.

Data regarding clinical and demographic properties and laboratory parameters were obtained from medical records. The study protocol was approved by the local ethics committee. This study was approved by a regional research ethics committee under the code IR.TBZMED.REC.1401.794.

Statistical Analysis

Data were reported as mean ± standard deviation (SD) or median (interquartile range) for parametric variables and percentages for categorical variables. The normality of continuous variables was confirmed by the Kolmogorov-Smirnov tests. Differences between groups were determined using the Mann-Whitney U test or independent t-test, as applicable; categorical variables were tested using Chi-square or Fisher exact tests, as applicable. Receiver operating characteristic curves were generated to determine MHR cutoff values for in-hospital mortality and MACEs. Univariate and multivariate binary logistic regression analyses were performed to identify independent predictors of in-hospital mortality and MACEs. Significance was set at P < 0.05. All statistical calculations were performed using SPSS software (version 16.0 for Windows, SPSS, Inc., Chicago, IL).

## Results

**Table:** Table are expressed as median and interquartile range (IQR) for parametric variables and percentage for categorical variables.

**Variables**	**Group 1** **n=326** **MHR<15.59**	**Group 2** **n=326** **MHR>15.59**	**P-value**
**Demographic**			
**Age (years)**	67.5 (55-77)	69(60-78)	0.01
**Male, n (%)**	189 (58)	189 (58)	1
**BMI, kg/m^2^**	24.2 (21.5-26.5)	24.2 (21.6-26.7)	0.26
**Diabetes mellitus, n (%)**	102 (31.3)	105 (32.2)	0.8
**Hypertension, n (%)**	144 (44.2)	165 (50.6)	0.1
**Hyperlipidemia, n (%)**	138 (42.3)	156 (47.9)	0.15
**Coronary Syndrome, n (%)**	92 (28.2)	86 (26.4)	0.59
**Family history of CAD, n (%)**	104 (31.9)	98 (30.1)	0.61
**Smoking, n (%)**	65 (19.9)	105 (32.2)	<0.001
**SBP (mmHg)**	110 (100-110)	110 (100-115)	0.52
**DBP (mmHg)**	70 (60-70)	70 (60-75)	0.63
**HR (bpm)**	84 (77-93)	85 (77-92)	0.78
**Laboratory parameters**			
**Hemoglobin (mg/dl)**	14.6 (12.8-15.9)	13.8 (12-15.5)	<0.001
**Platelet (/mm^3^)**	220 (140.6-292.2)	211 (148.3-308.2)	0.51
**WBC (10^3^ mL)**	9.2 (6.9-13.2)	15.1 (10.6-19.1)	<0.001
**Neutrophil (10^3^ mL)**	1.5 (0.9-3.6)	3.5 (1.75-5.72)	<0.001
**Lymphocyte (10^3^ mL)**	6.5 (4.4-9.4)	9.6 (6.56-12.8)	<0.001
**Monocyte (10^3^ mL)**	0.39 (0.27-0.49)	0.98(0.79-1.23)	<0.001
**Total cholesterol (mg/dl)**	185 (179-191)	186 (179-193)	0.35
**Triglyceride (mg/dl)**	170.33±16.15	170±17.01	0.8
**LDL (mg/dl)**	126 (120-136)	128 (119-137)	0.46
**HDL (mg/dl)**	41 (39-44)	41 (38-43)	0.07
**VLDL (mg/dl)**	15 (13-16)	15 (14-16)	0.83
**MHR**	9.65 (6.88-12.62)	23.6 (19.4-29.7)	<0.001
**Creatinine, mg/dL**	1.1 (0.9-1.3)	1.09 (0.9-1.3)	0.84
**Troponin I, ng/mL**	6.5 (5.37-9.6)	6.5 (5.5-8.3)	0.85
**CRP, mg/L**	3.3 (2.6-4.1)	3.3 (2.5-3.9)	0.31
**Killip class>1, n (%)**	260 (79.8)	267 (81.9)	0.05
**Angiographic Characteristics**			
**Culprit artery, n (%)**			
**LAD**	216 (66.3)	202 (62)	0.25
**CX**	134 (41.1)	137 (42)	0.81
**RCA**	137 (42)	134 (41.1)	0.81
**Multiple-vessel disease, n (%)**	222 (68.1)	259 (79.4)	<0.001
**LVEF, %**	37.5 (30-50)	35 (25-45)	0.05
**MACE, n (%)**	32 (9.8)	61 (18.7)	.001
**Mortality, n (%)**	8 (2.5)	29 (8.9)	<0.001

**Table:** Table **BMI**, body mass index; **CI**, confidence interval; **LVEF**, left ventricular ejection fraction; **MHR**, monocyte-to-high-density lipoprotein; **OR**, odds ratio.

**variables**	**Univariate**	**Multivariate**
OR	CI	P-value	OR	CI	P-value
**Age (years)**	1	0.97-1.02	0.97			
**Male, n (%)**	0.59	0.30-1.16	0.13			
**BMI, kg/m^2^**	**1.44**	**1.31-1.58**	**<0.001**	**1.44**	**1.30-1.59**	**<0.001**
**MHR**	**1.06**	**1.04-1.09**	**<0.001**	**1.05**	**1.02-1.08**	**0.002**
**Creatinine, mg/dL**	1.10	0.81-1.50	0.51			
**Troponin I, ng/mL**	0.94	0.85-1.03	0.23			
**Killip class>1, n (%)**	**1.70**	**1.17-2.46**	**.005**	**1.57**	**1.06-2.32**	**0.02**
**>1-vessel disease (multiple vessel diseases), n (%)**	**1.81**	**1.12-2.94**	**0.01**	1.56	0.88-2.79	0.12
**LVEF, %**	0.97	0.94-1.004	0.08			

**Table:** Table **BMI**, body mass index; **CI**, confidence interval; **LVEF**, left ventricular ejection fraction; **MACE**, major adverse cardiac events;** MHR, **monocyte-to-high-density lipoprotein; **OR**, odds ratio.

**variables**	**Univariate**	**Multivariate**
OR	CI	PV	OR	CI	PV
**Age (years)**	**.95**	**.93-.97**	**<0.001**	**.94**	**.92-.97**	**<0.001**
**Male, n (%)**	1.53	.96-2.44	.06			
**BMI, kg/m^2^**	**1.24**	**1.17-1.31**	**<0.001**	**1.28**	**1.20-1.37**	**<0.001**
**MHR**	**1.04**	**1.02-1.06**	**<0.001**	**1.05**	**1.02-1.08**	**<0.001**
**Creatinine, mg/dL**	**1.33**	**1.09-1.62**	**.005**	1.15	.87-1.52	.32
**Troponin I, ng/mL**	**1.18**	**1.13-1.24**	**<0.001**	**1.22**	**1.15-1.29**	**<0.001**
**Killip class>1, n (%)**	**1.29**	**1.02-1.62**	**.03**	1.30	.98-1.72	.06
**>1-vessel disease (multiple vessel disease), n (%)**	1.18	.87-1.61	.27			
**LVEF, %**	**.94**	**.92-.96**	**<0.001**	**.94**	**.91-.96**	**<0.001**

## Discussion

In this study, by analyzing the correlation between hospital admission MHR and in-hospital mortality and MACE in patients with STEMI who underwent primary PCI, we found that BMI, Killip>1, and MHR values were independently associated with in-hospital mortality, while age, BMI, troponin, LVEF, and MHR levels independently predicted MACE.

Atherosclerosis and plaque rupture due to STEMI are strongly associated with inflammation, which can be promoted by circulating monocytes. Oxidative stress and inflammation have been related to atherosclerotic progression and have become important factors in predicting adverse CAD outcomes [17][18][19][20]. Monocyte activation is an early step in the beginning of the atherosclerosis process, as evidenced by an increased count of circulating monocytes that is a predictor of plaque development. Endothelial cell dysfunction is the first stage of plaque development with mononuclear cells including monocytes and T lymphocytes attaching themselves to the endothelium. These cells in subendothelial space mature into macrophages and differentiate into foam cells by absorbing oxidized and other LDL-c. Foam cells release proinflammatory cytokines and tissue factor that increases the local inflammatory response, digests the internal elastic lamina and results in thrombus formation and ACS [21][22][23][24]. Tissue factor expression has been shown to increase following PCI, further demonstrating the pivotal role of monocytes in inflammation, thrombosis, and endothelial dysfunction [25]. Therefore, it is evident that macrophages and their precursor cells, monocytes, are key players in all stages of atherosclerosis and their involvement can lead to STEMI.

In contrast, HDL-c has well-known anti-inflammatory, antioxidant, and antithrombotic effects, particularly through suppressing monocyte activities and interrupting the differentiation of monocytes to macrophages, thus restricting the inflammatory response. HDL-c can also reduce the expression of adhesion molecules preventing monocyte recruitment to arterial walls [26][27]. In addition, research has shown that an increased level of HDL-c is associated with reduced extramedullary monocytepoiesis in ACSs, suppressing the production of various chemokines, and thus interrupting the monocyte production and differentiation process from the spleen - a major source of monocytes in ACS. Consequently, lower levels of HDL-c and LDL-c have been reported as independent predictors of mortality in ACSs and other clinical illness diseases [28][29]. 

In our study, serum cholesterol levels were lower in the MACE (+) group although this was not statistically significant and did not correlate with mortality and MACE in multivariate analysis. Nonetheless, HDL-c’s various effects highlight the importance of its role in cardiovascular diseases. MHR has emerged as an important indicator of inflammatory status and adverse cardiovascular outcomes, with its pioneering study conducted by Kanbay et al. [30]. They reported an association between increased MHR and the presence of cardiovascular comorbidities in chronic kidney disease patients [31]. After that, Canpolat et al. found that patients with pre-ablation high MHR values increased the risk of AF recurrence after ablation. Moreover, a study by MHR demonstrated that MHR was a significant predictor of stent thrombosis, mortality, and in-hospital MACE in patients with STEMI [32]. The association of monocytes and systemic inflammation, as well as the inhibitory effect of serum HDL-c on monocyte activation, suggested that an increased MHR may be predictive of adverse cardiovascular events in STEMI patients. Consequently, the monocyte-to-HDL-c ratio has been used as a new marker to predict clinical outcomes in several studies [16][33]. 

We also concluded that MHR is effective in predicting mortality and MACEs. In our study, receiver operating characteristic curve analysis revealed that MHR greater than 16.1 is a cutoff value for mortality and MHR greater than 17.96 is a cutoff value for MACE. The results of multiple studies also have illustrated that high MHR was associated with in-hospital mortality among STEMI patients who underwent primary PCI [16][30][33] . The levels of various laboratory parameters related to inflammation have been studied in individuals with cardiovascular disease and were found to be able to predict mortality and MACE. Furthermore, the admission troponin I level was identified as a predictor of negative cardiovascular outcomes. Our research revealed that higher admission troponin I levels were independently related to MACE.

## Conclusion

In this study demonstrated that admission MHR was an independent predictor of in-hospital mortality and MACE in patients with STEMI who underwent primary PCI. The results of this study suggest that MHR may be a useful biomarker for predicting adverse cardiovascular outcomes in patients with STEMI. Moreover, MHR can provide clinicians with additional information to facilitate the personalization of treatment strategies in these patients.

## Conflict of Interest

The authors declare that there is no conflict of interest.
